# Multi-Dimensional Wi-Fi Received Signal Strength Indicator Data Augmentation Based on Multi-Output Gaussian Process for Large-Scale Indoor Localization †

**DOI:** 10.3390/s24031026

**Published:** 2024-02-05

**Authors:** Zhe Tang, Sihao Li, Kyeong Soo Kim, Jeremy S. Smith

**Affiliations:** 1 School of Advanced Technology, Xi’an Jiaotong-Liverpool University (XJTLU), Suzhou 215123, China; zhe.tang15@student.xjtlu.edu.cn (Z.T.); sihao.li19@student.xjtlu.edu.cn (S.L.); 2 Department of Electrical Engineering and Electronics, University of Liverpool, Liverpool L69 3GJ, UK; j.s.smith@liverpool.ac.uk

**Keywords:** indoor localization, location fingerprinting, data augmentation, Multi-Output Gaussian Process (MOGP), regression, large-scale building complex

## Abstract

Location fingerprinting using Received Signal Strength Indicators (RSSIs) has become a popular technique for indoor localization due to its use of existing Wi-Fi infrastructure and Wi-Fi-enabled devices. Artificial intelligence/machine learning techniques such as Deep Neural Networks (DNNs) have been adopted to make location fingerprinting more accurate and reliable for large-scale indoor localization applications. However, the success of DNNs for indoor localization depends on the availability of a large amount of pre-processed and labeled data for training, the collection of which could be time-consuming in large-scale indoor environments and even challenging during a pandemic situation like COVID-19. To address these issues in data collection, we investigate multi-dimensional RSSI data augmentation based on the Multi-Output Gaussian Process (MOGP), which, unlike the Single-Output Gaussian Process (SOGP), can exploit the correlation among the RSSIs from multiple access points in a single floor, neighboring floors, or a single building by collectively processing them. The feasibility of MOGP-based multi-dimensional RSSI data augmentation is demonstrated through experiments using the hierarchical indoor localization model based on a Recurrent Neural Network (RNN)—i.e., one of the state-of-the-art multi-building and multi-floor localization models—and the publicly available UJIIndoorLoc multi-building and multi-floor indoor localization database. The RNN model trained with the UJIIndoorLoc database augmented with the augmentation mode of “by a single building”, where an MOGP model is fitted based on the entire RSSI data of a building, outperforms the other two augmentation modes and results in the three-dimensional localization error of 8.42 m.

## 1. Introduction

With the ever-increasing demand for Location-Based Service (LBS), localization based on various wireless technologies is subject to extensive research and development. The Global Navigation Satellite System (GNSS) provides reliable, real-time kinematic positioning and navigation in an outdoor environment with up to centimeter-level accuracy [1]. The GNSS, however, is not suitable for an indoor environment due to the blockage, attenuation, and scattering of satellite signals by the building structure and obstacles inside and outside the building [2]. Therefore, currently, indoor localization is mostly based on alternative technologies of infrared [3], ultrasonic [4], ultra-wideband (UWB) [5], ZigBee [6], Bluetooth [7], and Wi-Fi [8].

Of those wireless technologies for indoor localization, Wi-Fi is the most popular, as modern buildings are already equipped with a large amount of Wi-Fi infrastructure; therefore, indoor localization, based on Wi-Fi technology, does not incur an additional infrastructure overhead. Wi-Fi-based indoor localization methods can be grouped into two categories, i.e., those based on ranging and those based on location fingerprinting [9]. The ranging-based methods calculate the distance between a user and access points (APs) based on the received signal measurements—e.g., angles in the Angle of Arrival (AOA) and arrival times and their differences in Time of Arrival (TOA) and Time Difference of Arrival (TDoA) [10,11]—to estimate a user’s location via multilateration, which requires the exact locations of APs in advance and, if time measurements are involved, puts strict requirements on time synchronization among all devices. The fingerprinting-based methods, on the other hand, estimate a user’s location by comparing location fingerprints like Received Signal Strengths (RSSs) or Received Signal Strength Indicators (RSSIs) measured at the user’s current, unknown location during the online phase with those pre-collected during the offline phase at known Reference Points (RPs) in a location fingerprint database based on localization algorithms such as Deep Neural Networks (DNNs) (e.g., Feedforward Neural Networks (FNNs) [8] and Recurrent Neural Networks (RNNs) [12]) and the *k*-Nearest Neighbors (kNN) [13] algorithm. These methods do not require the locations of APs or strict time synchronization among the devices. Their localization performance, however, can be significantly affected by the number and the coverage of the location fingerprints measured at the RPs in the database, especially for a large-scale building complex [14].

In fact, the uneven spatial distribution of RPs is a major issue among the publicly available location fingerprint databases like UJIIndoorLoc [15], TUT [16], and WicLoc [17]; in the case of the UJIIndoorLoc, which is the most widely used multi-building and multi-floor RSSI database and has become a benchmark in the literature, the numbers of RPs are significantly different for the floors in the same building, and many fingerprint samples have spatial coordinates nearly identical to one another, indicating repeated samplings at the same RPs. These problems result in an inadequate spatial representation of data points and incomplete radio maps, which will be discussed in detail in Section 4.

To address these issues in fingerprint databases for large-scale multi-building and multi-floor indoor localization, in this paper, we propose methods for the multi-dimensional augmentation of fingerprint data based on the Multi-Output Gaussian Process (MOGP). The proposed multi-dimensional fingerprint data augmentation methods can improve the spatial coverage of data points in existing databases by generating synthetic fingerprint data at additional RPs, which could improve the localization accuracy of an indoor localization algorithm trained with the augmented database. It could also reduce the labor and time costs of constructing new databases using a well-prepared, but much-reduced, number of RPs.

The rest of the paper is organized as follows: In Section 2, we first review the dominant methods in data augmentation in general and proceed to the review of methods specific to indoor localization. In Section 3, we propose fingerprint data augmentation for large-scale multi-building and multi-floor indoor localization based on the MOGP and discuss the details of the proposed methods, including the selection of the kernel. Section 4 presents the results of our investigation of the effects of MOGP kernels and models with their hyperparameters and augmentation ratio on the performance of indoor localization using the UJIIndoorLoc database and the state-of-the-art DNN indoor localization model based on the hierarchical RNN [12]. Section 5 reviews the related work in comparison to our work. In Section 6, the conclusions are presented.

## 2. Related Work

In this section, we briefly review the basic principles of data augmentation in different research areas and the implementation of data augmentation specific to indoor localization.

### 2.1. Data Augmentation

The success of machine learning (ML) algorithms highly depends on the existence of a large number of datasets, but the collection of datasets, especially labeled ones for supervised learning, could be a challenging task in applications such as large-scale invasive examinations in medical testing [18,19] and multi-building and multi-floor indoor localization for a large-scale building complex [20] due to the issues of privacy and the high labor and time costs in collecting and labeling the data. Data augmentation has become a viable solution in this regard and has been applied widely to the categorization of images [21] and texts [22].

Image-based data augmentation algorithms can be grouped into image-processing-based or ML-based data augmentation methods: Image-processing-based data augmentation utilizes image processing techniques such as geometric transformations, flips, color transformations, cropping, and noise injection to augment data [21]. In the case of ML-based data augmentation, advanced ML algorithms like DNNs are used; a notable example is Generative Adversarial Networks (GANs), which have emerged as a representative approach to data augmentation using deep learning and have found a wide range of applications in areas such as medical imaging [18] and urban traffic control [23].

### 2.2. Indoor Localization Data Augmentation

RSSI or RSS values can be converted into a grayscale map or plotted as a radio map, enabling the application of the image-processing-based or ML-based data augmentation techniques mentioned in Section 2.1.

Sinha et al. converted a file containing 256 RSSI values into a 16×16 image as input to a Convolutional Neural Network (CNN) [24,25]. Lan et al. proposed a super-resolution-based fingerprint augmentation framework to achieve conversion between fingerprint data and fingerprint images [20].

Direct augmentation of indoor localization data using ML algorithms such as GANs is becoming popular. Njima et al. used a selective GAN to augment the UJIIndoorLoc database, and the localization prediction during the offline phase is demonstrated to significantly improve the localization accuracy [26]. Hilal et al. proposed DataLoc+ [27], a room-level data augmentation technique inspired by the dropout technique [28], to prevent overfitting. Rizk et al. used deep learning to implement data augmentation in cellular-based localization [29]. In [30,31], the authors used Single-Output Gaussian Process (SOGP) regression, also called Kriging in geostatistics, to augment the indoor localization data with a single building and single floor.

Note that there has been no prior work on the use of the MOGP to exploit the correlation among the RSSIs from multiple APs in multi-building and multi-floor indoor localization and investigate an optimal way of augmenting RSSI data based on MOGP, which is the major contribution of our work in this paper.

## 3. Multi-Dimensional Fingerprint Data Augmentation Based on MOGP

**Figure 1:**
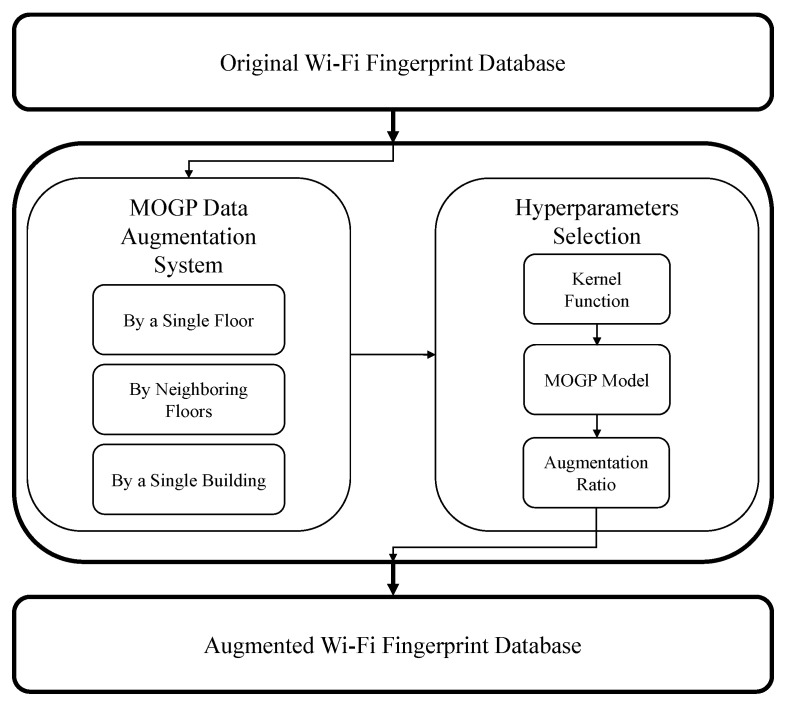
An overview of multi-dimensional fingerprint data augmentation based on MOGP.

Figure 1 shows an overview of the proposed multi-dimensional fingerprint data augmentation based on MOGP, which is applied to a fingerprint database constructed during the offline phase: To augment the fingerprint data, we first selected a data augmentation mode and then hyperparameters. The augmentation mode—i.e., “by a single floor”, “by neighboring floors”, or “by a single building”—determines the range of the existing fingerprint data to which an MOGP model is fitted; the hyperparameters, on the other hand, determine how to build an MOGP model and how to use the built MOGP model to augment the fingerprint data. After the multi-dimensional augmentation of fingerprint data based on the MOGP was completed, an indoor localization model was trained with both the original and the augmented fingerprint data.

### 3.1. Single-Output to Multi-Output Gaussian Process

Let D be a multi-building and multi-floor Wi-Fi fingerprint dataset of RSSI observations at *M* RPs, each of which consists of RSSIs from *N* APs, i.e.,

(1) $$D=\left(X,Y\right).$$

In (1), X is a collection of input vectors representing the location information of RPs—also called a design matrix in the literature [32]—and is given by:

(2) $$X=\left[x_{1},\ldots ,x_{M}\right]\in R^{4\times M},$$

and

(3) $$x_{i}={\left[B_{i},F_{i},X_{i},Y_{i}\right]}^{⊺},$$

where $B_{i}$ and $F_{i}$ are the building and the floor identifiers (IDs), and $X_{i}$ and $Y_{i}$ are the location coordinates of the *i*th RP, respectively; Y is a collection of output vectors representing the RSSIs measured at RPs and is given by:

(4) $$Y=\left[y_{1},\ldots ,y_{M}\right]\in R^{N\times M},$$

and

(5) $$y_{i}={\left[{\mathrm{RSSI}}_{i,1},\ldots ,{\mathrm{RSSI}}_{i,N}\right]}^{⊺},$$

where ${\mathrm{RSSI}}_{i,j}$ is the RSSI of the *j*th AP measured at the *i*th RP. In the case of the UJIIndoorLoc database, there are 19,938 RPs and 520 APs, which means *M* = 19,938 and N=520, and the *x* and *y* coordinates are according to the Universal Transverse Mercator (UTM) coordinate system in meters [15].

When we interpret an SOGP as a distribution of a function output f(x) for a given input x (i.e., the function-space view [32]), it can be directly described as follows:

(6) $$f\left(x\right)∼SOGP\left(m\left(x\right),k\left(x,x^{\prime }\right)\right),$$

where m(x) is the mean function, which, in practice, is typically set to zero, and $k(x,x^{\prime })$ is the covariance function, which is also called kernel. Unlike DNNs, the GP is a non-parametric model that can be interpreted as a union of a series of continuous random variables, each of which follows a Gaussian distribution. Note that, because an SOGP can handle a single output only, the data augmentation based on the SOGP is limited to the one-dimensional regression of RSSIs from one AP, which means that we need 520 SOGPs for the UJIIndoorLoc database with 520 APs.

Therefore, in the proposed data augmentation framework, we used an MOGP that can handle multiple outputs in an integrated way and thereby exploited the correlation among the RSSIs from multiple APs, which can be described as an extension of SOGP, i.e.,

(7) $$f\left(x\right)∼\mathrm{MOGP}\left(m\left(x\right),K\left(x,x^{\prime }\right)\right),$$

where

(8) $$f\left(x\right)={\left[f_{1}\left(x\right),\ldots ,f_{N}\left(x\right)\right]}^{⊺},$$

(9) $$m\left(x\right)={\left[m_{1}\left(x\right),\ldots ,m_{N}\left(x\right)\right]}^{⊺},$$

and

(10) $$K\left(x,x^{\prime }\right)=\left[\begin{array}{cccc} K_{1,1}\left(x,x^{\prime }\right) & K_{1,2}\left(x,x^{\prime }\right) & \cdots & K_{1,N}\left(x,x^{\prime }\right)\\ K_{2,1}\left(x,x^{\prime }\right) & K_{2,2}\left(x,x^{\prime }\right) & \cdots & K_{2,N}\left(x,x^{\prime }\right)\\ \vdots & \vdots & \ddots & \vdots \\ K_{N,1}\left(x,x^{\prime }\right) & K_{N,2}\left(x,x^{\prime }\right) & \cdots & K_{N,N}\left(x,x^{\prime }\right) \end{array}\right].$$

It is the extended kernel $K(x,x^{\prime })$ defined in (10) that enables an MOGP to take into account the correlation between multiple outputs (i.e., ${\left\{f_{j}\right\}}_{1\leq j\leq N}$), which is not possible with a group of independent SOGPs.

Now *N*-dimensional RSSI observations, which are noisy versions of the corresponding function values, can be modeled with independent and identically distributed Gaussian measurement noises as follows:

(11) y=f(x)+ϵ,

where

(12) $$\epsilon ∼N\left(0,\Sigma \right),$$

and

(13) $$\Sigma =\mathrm{diag}\left(\sigma_{1}^{2},\ldots ,\sigma_{N}^{2}\right).$$

In this case, the likelihood function is given by

(14) $$p\left(y|f,x,\Sigma \right)=N\left(f(x),\Sigma \right).$$

Given D in (1) as a training dataset, we can obtain the posterior distribution of the function value at a test point $x_{*}$ as follows:

(15) $$f\left(x_{*}\right)|X,Y,x_{*}∼N\left(\hat{f}{(x}_{*}),\Sigma_{*}\right),$$

where $\hat{f}$ and $\Sigma_{*}$ are the prediction mean and covariance, respectively; for details of their derivation in terms of (10) and (13) and the estimation of the covariance hyperparameters, readers are referred to [33].

**Figure 2:**
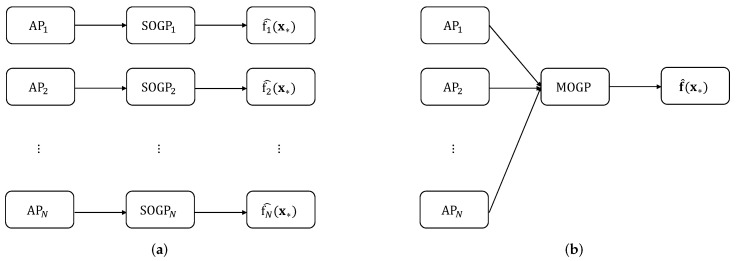
Block diagrams of fingerprint data augmentation based on (**a**) SOGP and (**b**) MOGP.

After post-processing (e.g., de-duplication and inverse normalization), the test point $x_{*}$ and the mean RSSI prediction $\hat{f}\left(x_{*}\right)$ are added to the collection of inputs and outputs (i.e., X and Y), respectively. Figure 2 highlights the difference between the SOGP and MOGP from the modeling point of view, where a single MOGP model handles the information on all APs in an integrated way.

### 3.2. Linear Models Based on Symmetric MOGP

MOGP models can be classified as symmetric or asymmetric. Symmetric MOGP is based on symmetric covariance functions to model symmetric correlations among output variables, which, for instance, results from the regression observations with independent and identically distributed Gaussian measurement noises described in (11)–(13). As the symmetric covariance functions of the symmetric MOGP can provide a simple model structure and thereby reduce the computational complexity in estimating hyperparameters, we focus on models based on the symmetric MOGP in the proposed data augmentation framework.

Of the symmetric MOGP models, the most widely used one is based on the Linear Model of Coregionalization (LMC), which captures the interactions among different outputs through a linear combination of latent functions: For j=1,…,N,

(16) $$f_{j}\left(x\right)=\sum_{q=1}^{Q}a_{j,q}u_{q}\left(x\right),$$

where $a_{j,q}$ is the coefficient for the latent function $u_{q}\left(x\right)$. The LMC can also be represented in a matrix form:

(17) f(x)=Au(x),

where

(18) $$A=\left[a_{i,j}\right]\in R^{N\times Q},$$

and

(19) $$u\left(x\right)={\left[u_{1}\left(x\right),\ldots ,u_{Q}\left(x\right)\right]}^{⊺}.$$

The latent functions ${\left\{u_{q}\right\}}_{1\leq q\leq Q}$, which are basis functions generating the outputs in GP regression, are SOGPs with zero mean and covariance defined by a kernel function that are independent of one another, i.e.,

(20) $$u_{q}\left(x\right)∼\mathrm{SOGP}\left(0,k_{q}\left(x,x^{\prime }\right)\right)\;\;\;\mathrm{for}\;q=1,\ldots ,Q,$$

and

(21) $$\mathrm{cov}\left(u_{q}\left(x\right),u_{q^{\prime }}\left(x^{\prime }\right)\right)=0\;\;\;\mathrm{for}\;q\neq q^{\prime }.$$

As for the *Q* value, Q=2 [34] or Q=N [35] has been suggested to improve the flexibility of the model and its ability to describe the differences in the data. Note that the special case of Q=1 is known as the Intrinsic Coregionalization Model (ICM).

### 3.3. Kernels

As discussed in Section 3.1, an MOGP model is completely specified by its kernel and mean function. The selection of a kernel, therefore, is critical in MOGP modeling, and here we discuss the characteristics of popular kernels with a focus on their ability to capture the correlation among individual data points, whose effects on indoor localization performance through data augmentation are investigated in detail in Section 4.

Kernels are mainly characterized by the three parameters of a variance ($\sigma^{2}$), a length scale (*l*), and a smoothness parameter (ν), though there are kernels that do not have those three parameters simultaneously. σ, also called a vertical scale, controls the vertical span of a kernel, *l* describes how quickly the correlation between two points drops as the distance between them increases, and ν determines whether a kernel is once differentiable or twice differentiable.

The most popular kernel is the Radial Basis Function (RBF), also known as the Gaussian kernel, which is defined by

(22) $$k_{\mathrm{RBF}}\left(x,x^{\prime }\right)=\sigma^{2}\exp\left(-\frac{∥x-x^{\prime }{∥}^{2}}{2l^{2}}\right).$$

The RBF kernel fits most input data because the correlation between individual data points in the domain is generally considered to decay smoothly as the distance between the data points increases [36]. Such a smooth decay, however, is not always the case; for example, in the case of a unit-step-like signal, the RBF kernel does not capture the characteristics of the signal at the moment of the jump accurately and tends to amplify the time of the signal change.

The Rational Quadratic (RQ) kernel is the mixture of RBF kernels with different length scales [37], which is defined by

(23) $$k_{\mathrm{RQ}}\left(x,x^{\prime }\right)=\sigma^{2}\exp{\left(1+\frac{∥x-x^{\prime }{∥}^{2}}{2\alpha l^{2}}\right)}^{-\alpha },$$

where α(>0) is the scale-mixture or form parameter. When α→∞, the RQ kernel becomes the RBF kernel [38]. However, it cannot solve the problem of excessive smoothness very well [39].

The Matérn family of kernels, on the other hand, can alleviate the oversmoothing at the moment of a signal jump [40]:

(24) $$k_{\mathrm{Matérn}}^{\nu }\left(x,x^{\prime }\right)=\sigma^{2}\frac{2^{1-\nu }}{\Gamma (\nu )}{\left(\frac{\sqrt{2\nu ∥x-x^{\prime }∥}}{l}\right)}^{\nu }K_{\nu }\left(\sqrt{2\nu ∥x-x^{\prime }∥}\right),$$

where Γ is a gamma function and $K_{\nu }$ is a modified Bessel function. $v=d+\frac{1}{2}$, where *d* is the order of a polynomial function. By setting ν to $\frac{3}{2}$ or $\frac{5}{2}$ and simplifying the general Matérn kernel function form (24) above, it is possible to obtain Matérn3/2 and Matérn5/2, which are:

(25) $$k_{\mathrm{Matérn}3/2}\left(x,x^{\prime }\right)=\sigma^{2}\left(1+\sqrt{3}\frac{|x,x^{\prime }|}{l}\right)\exp\left(-\sqrt{3}\frac{|x,x^{\prime }|}{l}\right),$$

and

(26) $$k_{\mathrm{Matérn}5/2}\left(x,x^{\prime }\right)=\sigma^{2}\left(1+\frac{\sqrt{5}\left|x,x^{\prime }\right|}{l}+\frac{5|x,x^{\prime }{|}^{2}}{3l^{2}}\right)\exp\left(-\frac{\sqrt{5}\left|x,x^{\prime }\right|}{l}\right).$$

These ν values also determine the smoothness of the kernel function. For example, the Matérn3/2 kernel function ($\nu =\frac{3}{2},\;d=1$) corresponds to a function that is once differentiable, while the Matérn5/2 kernel function ($\nu =\frac{5}{2},\;d=2$) corresponds to a function that is twice differentiable. By regulating ν, the Matérn class of kernel functions mitigate the over-smoothing problem of the RBF kernel in the signal mutation region because the RBF kernel function is infinitely differentiable.

Another solution to the oversmoothing of the RBF kernel is to replace the quadratic Euclidean distance with the absolute distance, which gives the Ornstein–Uhlenbeck (OU) kernel:

(27) $$k_{\mathrm{OU}}\left(x,x^{\prime }\right)=\sigma^{2}\exp\left(-\frac{∥x-x^{\prime }∥}{l}\right).$$

Note that the OU kernel is a special case of the Matérn kernel with $\nu =\frac{1}{2}$; refer to Section 4.2 of [32] for the details.

### 3.4. Data Augmentation Modes

Here, we describe in detail the three modes of data augmentation, which are shown in Figure 1, and discuss the scenarios suitable for each mode.

#### 3.4.1. By a Single Floor

**Figure 3:**
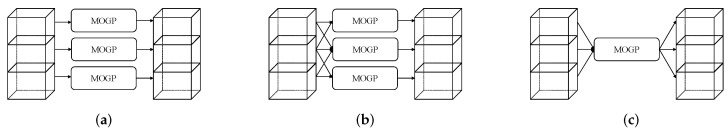
Three different modes of data augmentation: (**a**) by a single floor, (**b**) by neighboring floors, and (**c**) by a single building.

This data augmentation mode is the simplest of all as it fits an MOGP based only on the RSSIs from the APs on a single floor; sampling for the synthetic RSSI generation is also limited to the same floor as shown in Figure 3a. Therefore, compared to the other modes, it requires the smallest amount of data in fitting an MOGP model due to its simple structure. This mode is suitable for a floor with lower signal attenuation in the horizontal direction and higher signal attenuation or lower signal correlation (e.g., due to large building structure differences) in the vertical direction, the latter of which reduces the effects of APs located on different floors on the RSSIs of the floor under consideration. Unlike the SOGP limited to one AP in fitting, this mode still can take into account the effects of all the APs on the same floor.

#### 3.4.2. By Neighboring Floors

When the correlation among RSSIs from APs on neighboring floors is no longer negligible, the data augmentation can be extended to take into account the RSSIs from neighboring floors when generating synthetic RSSIs for a given floor as shown in Figure 3b, where the MOGP model for the second floor, for example, is fitted to the RSSIs from not only the second floor but also the first and the third floors. This would be the case especially when each floor of a building has a similar structure. Compared to the “by a single floor” mode, the kernel function of this mode needs an additional dimension for the floor height information.

#### 3.4.3. By a Single Building

In this mode, all the RSSIs of a building are considered as a coherent whole to be fitted by an MOGP model as shown in Figure 3c. The scenario suitable for this mode is that of a building with a large spatial variation with different architectural structures or signals on each floor.

## 4. Experimental Results

To investigate the effects of the MOGP-based Wi-Fi fingerprint data augmentation proposed in Section 3 on indoor localization performance, we used one of the state-of-the-art DNN models, which is based on a hierarchical RNN designed for large-scale multi-building and multi-floor indoor localization [12], together with the publicly available UJIIndoorLoc database [15]. The results of the performance evaluation of the proposed MOGP-based data augmentation are also compared with those of the state-of-the-art multi-building and multi-floor indoor localization schemes.

### 4.1. Experimental Setup

**Figure 4:**
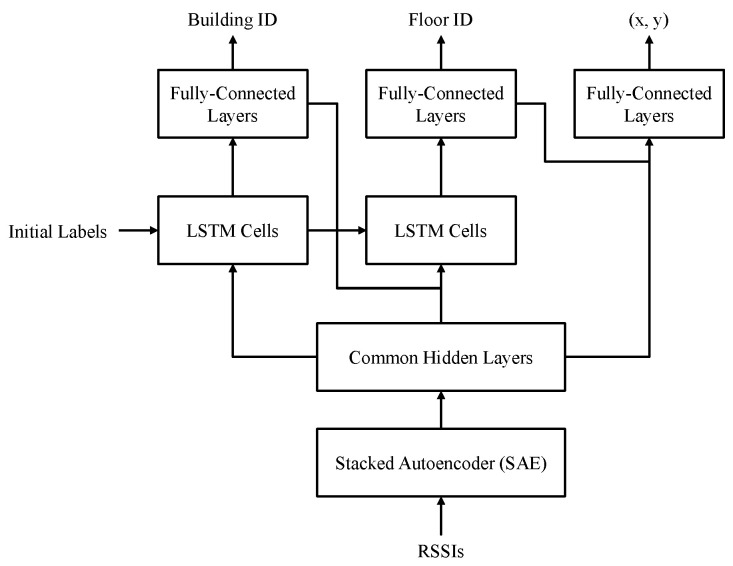
Network architecture of the RNN indoor localization model with LSTM cells [12].

Figure 4 shows the hierarchical RNN indoor localization model proposed in [12], which is used as a reference model for the evaluation of the localization performance of the proposed MOGP-based Wi-Fi fingerprint data augmentation system.

The Stacked Autoencoder (SAE) of the RNN model consists of three hidden layers of 256, 128, and 64 nodes, which are followed by two common hidden layers of 128 nodes. For building and floor classifiers, we have two stacked Long Short-Term Memory (LSTM) cells followed by two Fully Connected (FC) layers of 32 nodes and 1 output node. The position estimator consists of three FC layers of 512 and 512 nodes and 2 output nodes for floor-level two-dimensional (2D) coordinates [12]. We applied “early stopping” with a patience of 20 for the position estimator and 40 with “save best only” functions activated for the building and the floor classifiers. Table 1 summarizes the hyperparameter values for the experiments.

**Figure 5:**
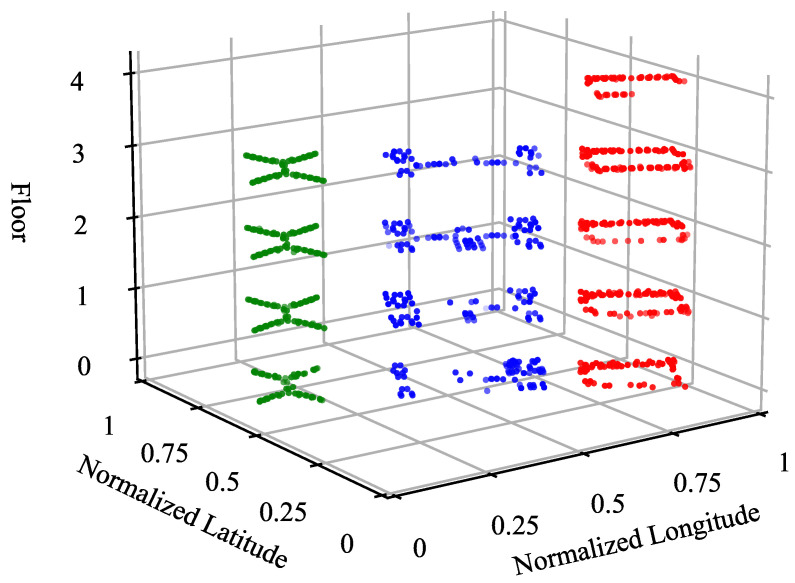
Spatial distribution of the RPs of the UJIIndoorLoc database over the buildings and the floors, where the green, the blue, and the red dots denote the RPs of Buildings 0, 1, and 2, respectively.

Table 2 summarizes the number of RPs on each floor of the three buildings of the UJIIndoorLoc Wi-Fi fingerprint database, which shows that the numbers of per-floor RPs are quite different from one another even within the same building: In Building 2, for example, the number of RPs on Floor 3 is about 2.5 times that on Floor 4 (i.e., 2709 vs. 1102). The uneven spatial distribution of the RPs within the same building is more clearly visualized in Figure 5, where the coordinates are normalized for the area covering the three buildings.

Note that because the publicly available UJIIndoorLoc database includes only training and validation datasets but not a testing dataset, the latter of which was provided only to the competitors at the Evaluating Ambient Assisted Living (EvAAL) competition at the International Conference on Indoor Positioning and Indoor Navigation (IPIN) 2015 [41], we split the training dataset into new training and validation datasets with the ratio of 70:30 for training and validation and used the validation dataset as a new testing dataset like the performance evaluation of most of the multi-building and multi-floor indoor localization schemes based on the UJIIndoorLoc database in the literature [8].

The MOGP regression for the proposed data augmentation was implemented using GPy [42] per the steps outlined in Section 3.1, and all the experiments described in Section 4.2.1, Section 4.2.2, Section 4.2.3, Section 4.2.4 and Section 4.2.5 were run on a workstation with an Intel Core i9-9900X processor, two Nvidia GeForce RTX 2080 Ti graphics cards, and 32 GB of RAM with the default parameters summarized in Table 3, where the augmentation ratio is defined by

(28) $$r=\frac{\mathrm{Number}\;\mathrm{of}\;\mathrm{Augmented}\;\mathrm{Data}}{\mathrm{Number}\;\mathrm{of}\;\mathrm{Original}\;\mathrm{Data}}.$$

### 4.2. Effects of the Proposed MOGP-Based Data Augmentation on Indoor Localization Performance

Here, we investigate the effects of the various components and hyperparameters of the proposed MOGP-based data augmentation—i.e., data augmentation modes, MOGP models, augmentation ratios, and kernels and their hyperparameters—on the indoor localization performance. In the following subsections, we use the default parameter values summarized in Table 3 unless explicitly mentioned otherwise.

#### 4.2.1. Data Augmentation Modes

Table 4 summarizes the three-dimensional (3D) localization errors ([41], Equation (2)) of the three data augmentation modes discussed in Section 3.4.

It is clear from the results that, of the three modes, the data augmentation mode of “by a single building” provides the best performance under the multi-building and multi-floor environment of the UJIIndoorLoc database because its kernel function includes the vertical dimension as well as the horizontal ones and thereby can fully take into account the correlation among all RSSI data over the whole building. These results also demonstrate that the effects of the APs located on different floors—including those on non-neighboring floors—on the augmentation cannot be ignored.

#### 4.2.2. Number of LMC Latent Functions

The effects of the number of LMC latent functions *Q* on the localization performance are summarized in Table 5. Except for the case of Q=4, the localization error decreases as *Q* increases. Given the huge computational complexity resulting from the use of a large number of latent functions, however, we would choose Q=2 in practice, to strike a balance between performance and computational complexity, which is also in line with the suggestions from the literature [34,35].

#### 4.2.3. Augmentation Ratio

Given the uneven spatial distribution of the RPs—even the complete absence of the RPs in some areas—in the UJIIndoorLoc database, it is worthwhile to investigate the effect of the data augmentation ratio *r*. Though excessive data augmentation with a large value of *r* significantly increases the amount of total data for training, it would result in ignorance of the features of the original data. A small augmentation ratio, on the other hand, may not be able to address the issue of an uneven spatial distribution. Table 6 shows the 3D localization errors for different augmentation ratios, where the case of r=1 provides the best result.

#### 4.2.4. Kernels

**Figure 6:**
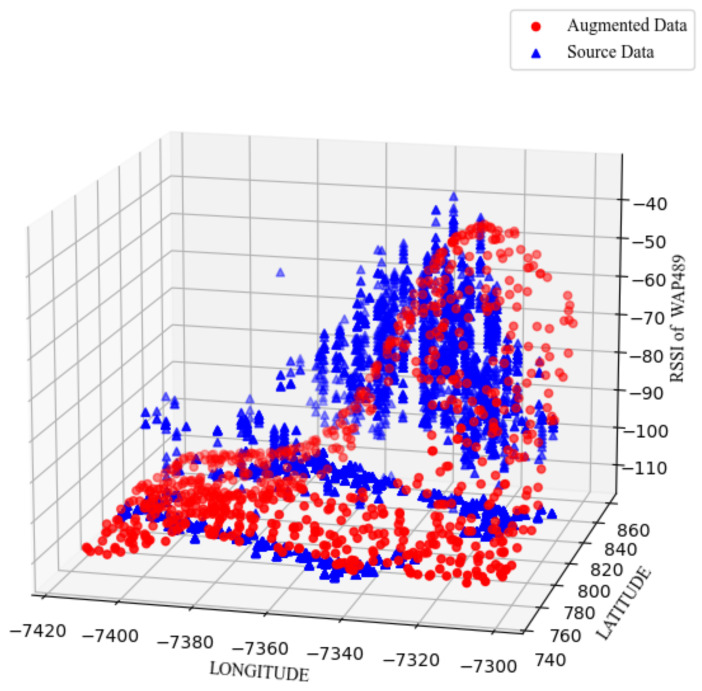
MOGP-based data augmentation of the RSSIs from WAP489 of the UJIIndoorLoc database based on the Matérn5/2 kernel with the parameters in Table 3.

The importance of different kernels in MOGP modeling is discussed in Section 3.3, and their effects on the localization performance are summarized in Table 7. Of the kernels under consideration, Matérn5/2 provides the best performance. For ease of visualization, we selected a single AP out of the 520 APs of the UJIIndoorLoc database (i.e., WAP489) and show its original RSSIs and the augmented RSSIs based on them together in Figure 6. From the results, we observe that the MOGP model smooths the fluctuations of the original RSSIs, which implies that the MOGP model considers some extreme points of the original data as noises.

#### 4.2.5. Kernel Hyperparameters

As discussed in Section 3.3, kernels have two basic hyperparameters of a variance $\sigma^{2}$ (also called a scale factor) and a length scale *l*. Table 8 and Table 9 summarize the 3D localization errors for different values of variance and length scale for the Matérn5/2 kernel, respectively, where $\sigma^{2}=1$ and l=10 provide the best performance.

The variance $\sigma^{2}$ scales the kernel and controls the spread of samples from the mean to a certain extent; therefore, a larger variance can alleviate the problem of oversmoothing in data augmentation. The length scale *l*, on the other hand, controls the extrapolation capability of the model or defines the limiting distance to which the maximum predictable belongs. Note that deciding the values of kernel hyperparameters for given data and application scenarios remains an open issue.

### 4.3. Comparison with the State of the Art

Table 10 summarizes the multi-building and multi-floor indoor localization performance of some of the state-of-the-art schemes [41] as well as the hierarchical RNN [12] with and without the proposed MOGP-based data augmentation using the default parameters in Table 3; in addition to 3D error, building hit rate and floor hit rate are provided as performance metrics, which are defined as a rate of correct identification of building ID and that of floor ID, respectively.

The results of the four schemes discussed as part of the 2015 EvAAL/IPIN competition in [41]—i.e., MOSAIC, HFTS, RTLS@UM and ICSL—are based on the training, the validation, and the test dataset of the UJIIndoorLoc database, the last of which is not publicly available. Furthermore, the four schemes are not as scalable as the schemes based on a single DNN. The comparison between the results of the four schemes and those of the hierarchical RNN with and without the proposed MOGP-based data augmentation are presented in Table 10 and is therefore not fair but could be used as a relative indicator of the performance of the proposed scheme.

The comparison with the hierarchical RNN without data augmentation shows that the proposed MOGP-based data augmentation reduces the 3D error by 0.2 m while slightly decreasing the floor hit rate, and this demonstrates its feasibility in multi-building and multi-floor indoor localization. Note that the only metric defined for the EvAAL/IPIN competition based on the UJIIndoorLoc database was the 3D error, which already takes into account the effects of the building and the floor hit rates in terms of penalties [41].

**Figure 7:**
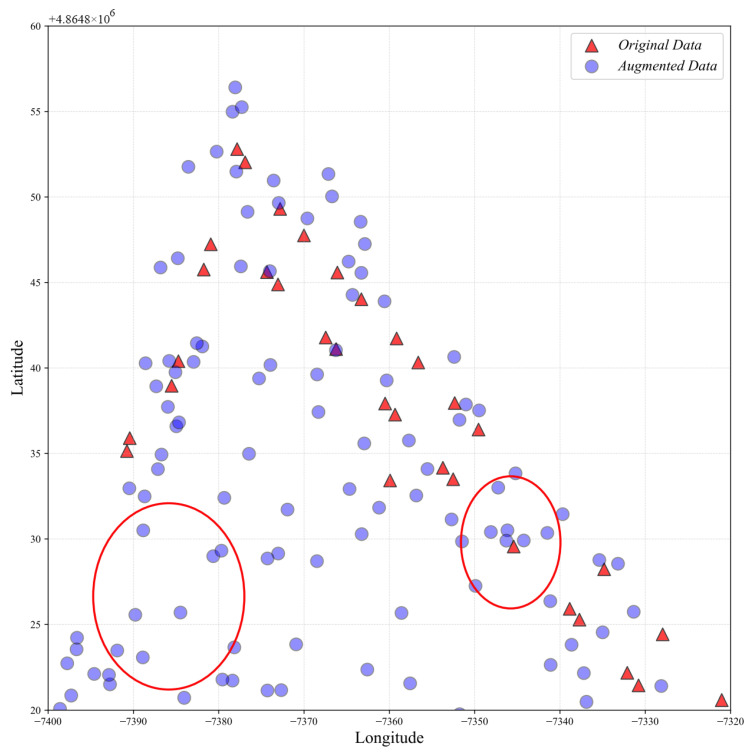
Spatial distribution of the original and the augmented RSSIs for the corner of the fourth floor of Building 2 of the UJIIndoorLoc database, where the red circles indicate two potential problems of the lack of original RSSI data and insufficient RP coverage.

Figure 7 could explain why the proposed MOGP-based data augmentation can improve the localization performance of the hierarchical RNN, where the red triangles and the blue dots indicate the RPs of the original and the augmented RSSIs, respectively, for the corner of the fourth floor of Building 2 of the UJIIndoorLoc database; the sampling of latitude and longitude for the augmentation is based on a Gaussian distribution.

In Figure 7, the two red circles highlight the regions poorly covered by the original RSSIs, with the left one being an extreme case of no coverage at all; the poor coverage results from the difficulties in accessing rooms like personal offices. The augmented RSSIs, on the other hand, successfully fill the poorly covered areas, which demonstrates the feasibility of the proposed MOGP-based data augmentation in improving the spatial coverage of the RSSI data.

It is worth noting that, due to the lack of detailed building coordinates and internal floor structure maps for the UJIIndoorLoc database, the sampling of latitude and longitude cannot fully take into account the building and floor structures, which limits the positioning accuracy of the augmented data.

## 5. Comparison to Related Work

In this section, we provide a qualitative comparison between the proposed Wi-Fi fingerprint data augmentation scheme based on the MOGP and the state-of-the-art ones based on DNNs, which is the best possible comparison given the lack of source code for implementation and the differences in underlying datasets and evaluation metrics (e.g., localization accuracy vs. RSSI value error) among them. In this regard, we mainly focus on the model interpretability, the localization type, and the localization effect of each augmentation scheme.

*Model interpretability*: The major difference between MOGP-based and DNN-based schemes is that an MOGP, which is a special case of a GP, can be completely defined by just two functions—i.e., a kernel and a mean—unlike a DNN based on a typically large number of weights, biases, and nonlinear activation functions; compared to a DNN that has been considered a black box due to the difficulty of understanding the inner workings of the model from input to output, an MOGP has a higher degree of interpretability because outputs are weighted combinations of inputs in the data space. Therefore, an MOGP-based model allows better traceability of each augmentation point and ability to modify observations by changing the local distribution, which makes it easier to understand and control the model with the kernel function. As discussed in Section 4.2.5, however, tuning kernel hyperparameters for given data and application scenarios still remains an open issue.

*Localization type*: The type of localization ranges from single-floor to multi-floor within a single building to multi-building and multi-floor, the latter two of which require not only more complicated fingerprint databases but also more advanced augmentation schemes for the estimation of 3D locations. It is interesting in this regard that, although the proposed MOGP-based data augmentation scheme, s-GAN [26], and DL Augmentation [25] methods use the multi-building and multi-floor UJIIndoorLoc database, the MOGP-based data augmentation scheme is the only one that provides the results of the evaluation of multi-building and multi-floor localization performance based on the full datasets of the UJIIndoorLoc database. DataLoc+ [27], on the other hand, uses the fingerprint data measured on a single floor of a hospital, which reflects many devices and the movement of people carrying them in the hospital. In the cases of the CAN [43], DL Approach [44], and Between-Location [45] methods, small-scale, proprietary, single-floor databases are used, where it would be easier to obtain the details of the internal building structure and choose the optimal locations of APs and RPs based on them for the improvement of the stability of radio maps; in these cases, the results presented in the papers cannot be reproduced by other researchers.

Table 11 summarizes our discussion of the qualitative comparison of the proposed and the state-of-the-art DNN-based data augmentation schemes for indoor localization.

As for the s-GAN [26], because it only provides the results of single-floor data augmentation and localization for Building 1 Floor 2 of the UJIIndoorLoc database, we also applied the proposed MOGP-based data augmentation for the same building and floor and obtained the 2D localization error using the hierarchical RNN for comparison, as summarized in Table 12.

Unlike the proposed scheme, the s-GAN uses a GAN to generate augmented RSSI data, associates pseudo-labels with the generated data using semi-supervised learning, and filters out inappropriate augmented RSSI data before location estimation. Note that the results shown in Table 12 are not based on identical conditions. The data augmentation for the s-GAN is based only on 190 APs out of the 520 APs of the UJIIndoorLoc database. The s-GAN also filtered out unnecessary and inaccurate augmented RSSI data, during which the s-GAN had to generate a large amount of augmented data, i.e., more than 40 times as much as the original data [26]. The proposed MOGP-based data augmentation, on the other hand, uses all 520 APs and feeds all the augmented data to the localization network without filtering.

In summary, the major advantages of the proposed MOGP-based data augmentation scheme over DNN-based ones are its higher interpretability and ability to achieve a localization performance comparable to or even better that that of DNN-based ones without complicated pre-processing and filtering, which could make the model structure simpler and more intuitive.

## 6. Conclusions

In this paper, we have proposed using multi-dimensional fingerprint data augmentation for indoor localization in a large-scale building complex based on MOGP and systematically investigated the effects of the various aspects of MOGP-based data augmentation on localization performance.

Through the extensive experiments using the-state-of-the-art DNN indoor localization model based on the hierarchical RNN [12] and the UJIIndoorLoc database [15], we first investigated the effects of MOGP kernels and their hyperparameters on the localization performance and found that Matérn5/2 with a variance of 1 and the length scale of 10 provides the best performance in the case of a single kernel. As for the MOGP models, we focused on the effect of the number of the latent function *Q* of LMC (with ICM being the special case of LMC with Q=1) and found that the localization error becomes minimum when *Q* is equal to the number of MOGP outputs *N* of the UJIIndoorLoc database; we also found that Q=2 can provide decent localization performance (i.e., slightly worse than Q=3 and better than Q=4 as shown in Table 5) and reached the right balance between localization performance and computational complexity as suggested in [34,35].

The effect of the data augmentation ratio was also investigated in order to explore the extent to which we can augment a fingerprint database without significantly altering the statistical characteristics of the original data. The experimental results show that we can generate synthetic RSSI data up to ten times the original data—i.e., the augmentation ratio of 10—through the proposed multi-dimensional MOGP-based data augmentation with localization performance nearly as good as that of the original data without augmentation. This result is important because it means that we can extend the spatial coverage of the combined RPs of a fingerprint database using the proposed MOGP-based data augmentation and thereby could improve the localization performance at the locations that are not part of the training dataset.

During our investigation of the effects of various aspects of MOGP-based data augmentation on localization performance, we focused our investigation of MOGP on the linear models of ICM and LMC and based the experiments only on the UJIIndoorLoc database. Our investigation in this paper, therefore, could be extended to other MOGP models with kernels better suited for indoor localization and multi-building and multi-floor databases (e.g., [46,47]).

One important issue in the indoor localization research based on the existing fingerprint databases is the inadequate consideration of interference factors, which are often time-varying: In large shopping malls and transport hubs, dense crowds of moving people are the main interference, while in underground car parks a large number of temporary APs are the main interference. Fingerprint data augmentation taking into account those time-varying interference factors, therefore, is another interesting topic for further research.

## Figures and Tables

**Table 1:** Hyperparameters and their values in the RNN model.

Parameter	Value
SAE Hidden Layers	256-128-64
SAE Activation	ReLU
SAE Optimizer	Adam
SAE Loss	MSE
Common Hidden Layers	128-128
Common Activation	ReLU
Common Dropout	0.2
Common Loss	MSE
LSTM Cells	256-512
LSTM Activation	ReLU
LSTM Optimizer	Adam
LSTM Loss	MSE
Building/Floor Classifier Hidden Layers	32-1
Building/Floor Classifier Activation	MSE
Building/Floor Classifier Optimizer	Adam
Building/Floor Classifier Dropout	0.2
Building/Floor Classifier Loss	ReLU
Position Estimator Hidden Layers	512-512-2
Position Estimator Activation	MSE
Position Estimator Optimizer	Adam
Position Estimator Dropout	0.1
Position Estimator Loss	tanh

**Table 2:** Number of per-floor RPs over the three buildings of the UJIIndoorLoc database.

	Building 0	Building 1	Building 2
Floor 0	1059	1368	1942
Floor 1	1356	1484	2162
Floor 2	1443	1396	1577
Floor 3	1391	948	2709
Floor 4	N/A	N/A	1102
Total	5249	5196	9492

**Table 3:** Default parameter values for the MOGP-based multi-dimensional augmentation of fingerprint data.

Parameter	Value
Data Augmentation Mode	By a single building
Augmentation Ratio (*r*)	1
Number of Latent Functions (*Q*)	*N*
Kernel	Matérn5/2
Variance ($\sigma^{2}$)	1
Length scale (*l*)	10

**Table 4:** 3D localization error by different data augmentation modes.

Data Augmentation Mode	3D Error [m]
By a single floor	8.67
By neighboring floors	8.60
By a single building	8.42

**Table 5:** 3D localization error by different numbers of latent functions in LMC.

Numbers of Latent Functions (*Q*)	3D Error [m]
1	8.70
2	8.60
3	8.58
4	8.61
*N*	8.42

**Table 6:** 3D localization error by different augmentation ratios.

Augmentation Ratio	0 *	0.5	1	5	10
3D Error [m]	8.62	8.72	8.42	8.69	8.88

* Based on the unaugmented data [12].

**Table 7:** 3D localization error by different kernels.

Kernel	RBF	RQ *	Matérn3/2	Matérn5/2	OU
3D Error [m]	8.96	9.17	8.78	8.42	8.86

* α=2.

**Table 8:** 3D localization error by different values of the variance of the Matérn5/2 kernel.

Variance ($\sigma^{2}$)	0.1	1	10
3D Error [m]	8.80	8.42	8.69

**Table 9:** 3D localization error by different values of the length scale of the Matérn5/2 kernel.

Length Scale (*l*)	1	10	100
3D Error [m]	8.78	8.42	8.83

**Table 10:** Multi-building and multi-floor indoor localization performance of the proposed and the state-of-the-art schemes.

Localization Scheme	Building Hit Rate [%]	Floor Hit Rate [%]	3D Error [m]
**Proposed** *	**100** ^†^	94.20	8.42
Hierarchical RNN [12]	**100**	95.23	8.62
MOSAIC [41]	98.65	93.86	11.64
HFTS [41]	**100**	**96.25**	8.49
RTLS@UM [41]	**100**	93.74	**6.20**
ICSL [41]	**100**	86.93	7.67

* Hierarchical RNN [12] with the proposed MOGP-based data augmentation using the default parameters in Table 3. ^†^ The numbers in bold are the best results in each performance measure.

**Table 11:** Comparison of data augmentation schemes for indoor localization.

Augmentation Scheme	Model Interpretability	Localization Type	Notes
**Proposed**	High	Multi-Building Multi-Floor	MOGP
s-GAN [26]	Low	Single-Floor	GAN
DataLoc+ [27]	Low	Single-Floor	Dropout
DL Augmentation [25]	Low	Single-Floor	Deep Learning
CAN [43]	Low	Single-Floor	Conditional Adversarial Networks
DL Approach [44]	Low	Single-Floor	AlexNet
Between-Location [45]	Low	Single-Floor	Between-Class Learning

**Table 12:** Comparison with the s-GAN using Building 1 Floor 2 of the UJIIndoorLoc database.

Augmentation Scheme	Localization Error [m]	Improvement [m, %]
s-GAN [26]	4.1	-
s-GAN with Augmentation * [26]	3.47	0.63, 15.36
Hierarchical RNN [12]	4.2	-
Hierarchical RNN [12] with MOGP-based Augmentation ^†^	**3.40** ^‡^	**0.80**, **19.04**

* Based on 190 APs of the 520 APs of the UJIIndoorLoc database. ^†^ Based on the 520 APs of the UJIIndoorLoc database using the default parameters in Table 3. ^‡^ The numbers in bold are the best results in each performance measure.

## Data Availability

No new data were created or analyzed in this study. Data sharing is not applicable to this article.
